# Investigating mouse superior colliculus function using two-photon calcium imaging

**DOI:** 10.3389/fncel.2026.1922414

**Published:** 2026-10-01

**Authors:** Haesuk Lee, Greg J. Stuart

**Affiliations:** Department of Physiology, Neuroscience Program, Biomedicine Discovery Institute, Monash University, Clayton, VIC, Australia

**Keywords:** calcium imaging, methods, neuron, superior colliculus, two photon imaging

## Abstract

*In vivo* two-photon calcium imaging has fuelled ongoing progress in understanding the superior colliculus (SC). This has been achieved through technical endeavors that have made this midbrain structure optically accessible. In this mini–review, we summarize these methods. New techniques, materials, and ideas will further advance these methods, making SC imaging both less invasive and more accessible in the future. In doing so, two-photon calcium imaging will undoubtedly continue to contribute to our understanding of the SC and the cellular and circuit mechanisms underlying sensory processing and sensorimotor function in the brain.

## Introduction

Detecting salient stimuli in the environment and generating appropriate motor responses are essential for survival. The superior colliculus (SC), an evolutionarily conserved midbrain structure, is crucial for such sensorimotor transformations. For example, in mice, the SC is involved in coordinating visually guided approach and avoidance behaviors, supporting prey capture and predator evasion (Campagner et al., 2023; Comoli et al., 2012; Hoy et al., 2019; Shang et al., 2015; Wei et al., 2015). Other studies indicate that the SC is also involved in more complex cognitive functions, such as decision-making and reward-related behaviors (Cun et al., 2025; Wang et al., 2020). Consistent with a role in sensorimotor transformations, the superficial layers of the SC receive direct projections from the retina (Ellis et al., 2016), with its deeper layers receiving visual, auditory, and somatosensory inputs (Doykos et al., 2020; May, 2006).

While pioneering studies have primarily used electrophysiological techniques to understand the properties of different cell types in the SC and their function (Gale and Murphy, 2014, 2016; Wang et al., 2010), the application of optical methods, such as two-photon calcium imaging, offers distinct advantages for studying SC function. Notably, the high spatial resolution of two-photon calcium imaging enables the mapping of functional organization at the single-cell or even subcellular level, offering a powerful means of investigating neural computations in the intact brain (Grienberger et al., 2022). However, limited optical access presents a major obstacle to performing *in vivo* two-photon calcium imaging in the intact mouse SC. Although the mouse SC sits on the roof of the midbrain, its anterior part is covered by the overlying visual cortex, while its posterior portion is positioned beneath the transverse sinus (TS). As a result, while optical access to cortical areas can be obtained by performing a craniotomy directly above the target cortical region (Augustinaite and Kuhn, 2020), additional surgical interventions are necessary to access the SC.

To date, several approaches have been developed to address this technical issue. These approaches have allowed researchers to successfully perform two-photon calcium imaging in the SC of both acute and chronic preparations for up to 6 months (Table 1). However, the approaches used are technically challenging and have distinct advantages and disadvantages. In this review, we discuss the advantages and disadvantages of the different methods currently used to gain optical access to the mouse SC. For a more comprehensive overview of the SC, we direct readers to in-depth reviews by others (Basso et al., 2021; Cang et al., 2024; Cang et al., 2018; Ito and Feldheim, 2018).

**Table 1:** Published studies using two-photon calcium imaging in the mouse SC.

**Paper**	**Calcium indicator**	**Target**	**Acute/chronic**	**Imaging depth**
Method 1: Removal of the overlying cortex				
Inayat et al. (2015)	Cal-520 AM	SC neurons	Acute	Superficial SC
Kasai and Isa (2016)	OGB-1 AM	SC neurons	Acute	80–250 μm
Shi et al. (2017)	Cal-520 AM	SC neurons	Acute	Superficial SC
Barchini et al. (2018)	Cal-520 AM AAV2/1-Syn-H2B-GCaMP6s	SC neurons	Acute	Cal-520: up to 50 μm GCaMP6s: up to 205 μm
de Malmazet et al. (2018)	AAV2/1-Syn-GCaMP6s	SC neurons	Chronic; up to 3 months	50–400 μm
Kasai and Isa (2022)	AAV1-hSyn-GCaMP6f AAV1-hSyn-Flex-GCaMP6f	SC neurons Vgat+ neurons	Chronic	10–245 μm
Li C. et al. (2023)	AAV1-EF1α-fDIO-GCaMP6s	Gad2 + neurons	Chronic	Up to 300 μm
Kuhn et al. (2025)	AAV1-hSyn-Flex-GCaMP6s	Ntsr1 + neurons	Chronic	0–250 μm
Method 2: Displacement of the transverse sinus				
Feinberg and Meister (2015)	AAV2/1-hSyn1-GCaMP6s	SC neurons	Chronic; Up to 6 months	50–300 μm
Savier et al. (2019)	AAV1-Syn-GCaMP6f	SC neurons	Chronic; Up to 2 months	Up to 50 μm
Li et al. (2020)	AAV2/1-hSyn1-GCaMP6f	SC neurons	Chronic; Up to 6 weeks	Up to 350 μm
Schroder et al. (2020)	AAV2/1-Syn-GCaMP6f AAV2/2-hSyn1-SyGCaMP6f	SC neurons Retinal boutons	Chronic	15–100 μm
Chen et al. (2021)	AAV1-Syn-GCaMP6f	SC neurons	Chronic	Up to 50 μm
Baruchin et al. (2023)	AAV2/1-Syn-GCaMP6f	SC neurons	Chronic	12–260 μm
Li and Meister (2023)	AAV2/1-hSyn1-GCaMP6f AAV2/1-Syn-Flex-GCaMP6f	SC neurons Vglut2+, Vgat+, Tac1+, Rorb+, Ntsr1 + neurons	Chronic; Up to 2 months	Up to 350 μm
Liu et al. (2023)	AAV1-Syn-GCaMP6f	SC neurons	Chronic	Up to 300 μm
Molotkov et al. (2023)	AAV2/9 or AAV1/2–hSyn–jGCaMP7f, jGCaMP8m or jRGECO1a AAV1/2–hSyn–axon-GCaMP6s	SC neurons Retinal axons	Chronic	120–220 μm
Li (2025)	AAV2/1-hSyn1-GCaMP6f AAV2/1-Syn-Flex-GCaMP6f	SC neurons Vglut2+, Vgat+, Tac1+, Rorb+, Ntsr1 + neurons	Chronic	Up to 350 μm
Relota et al. (2025)	AAV9-Syn-Flex-GCaMP6s	Ntsr1 + neurons	Chronic	300–400 μm
Wu et al. (2025)	AAV2/9-Syn-jGCaMP8m	SC neurons	Chronic	Up to 350 μm
Both methods				
Liang et al. (2023)	AAV2/1-Syn-GCaMP6s AAV2/1-Syn-H2B-GCaMP6s AAV2/1-Syn-Flex-GCaMP6s	SC neurons Gad2 + neurons	Chronic	Up to 180 μm
DePiero et al. (2024)	AAV1-hSyn-GCaMP6f	SC neurons	Chronic	Superficial SC

Studies are grouped according to the surgical approach used to access the SC and are ordered chronologically within each category. In the target column, “SC neurons” indicates non-specific labeling of SC neurons. In the acute/chronic column, “chronic” without a specified duration indicates that a chronic preparation was used, but the exact duration was not reported. Imaging depths are reported relative to the SC surface.

## Method 1: Removal of the overlying cortex

As mentioned above, the anterior part of the mouse SC is covered by the visual cortex, while its posterior part lies underneath the transverse sinus. Hence, the overlying structures must be either removed or displaced to gain optical access to the underlying SC. The first method, arguably the most primitive yet direct, is to remove the overlying visual cortex. First implemented in early imaging studies, many researchers have applied this approach to perform two-photon calcium imaging in the mouse SC.

With this approach, a 2–3-mm-diameter craniotomy is performed above the stereotaxic coordinates of the SC, followed by removal of the dura. Next, the visual cortex above the SC is manually removed via gentle aspiration under visual guidance until the SC is exposed. For aspiration, an 18-gauge needle coupled to a suction line can be used (Inayat et al., 2015). This procedure must be performed very carefully to minimize damage to the underlying SC, as well as to nearby vasculature, including both the sagittal sinus and the transverse sinus. Once the SC is successfully exposed, a cranial imaging window through which two-photon calcium imaging can be performed is implanted above the SC and fixed with dental cement or cyanoacrylate glue (Figure 1). Imaging windows typically consist of a glass assembly with a thickness of 1 to 3 mm (de Malmazet et al., 2018; Kasai and Isa, 2022).

**Figure 1:**
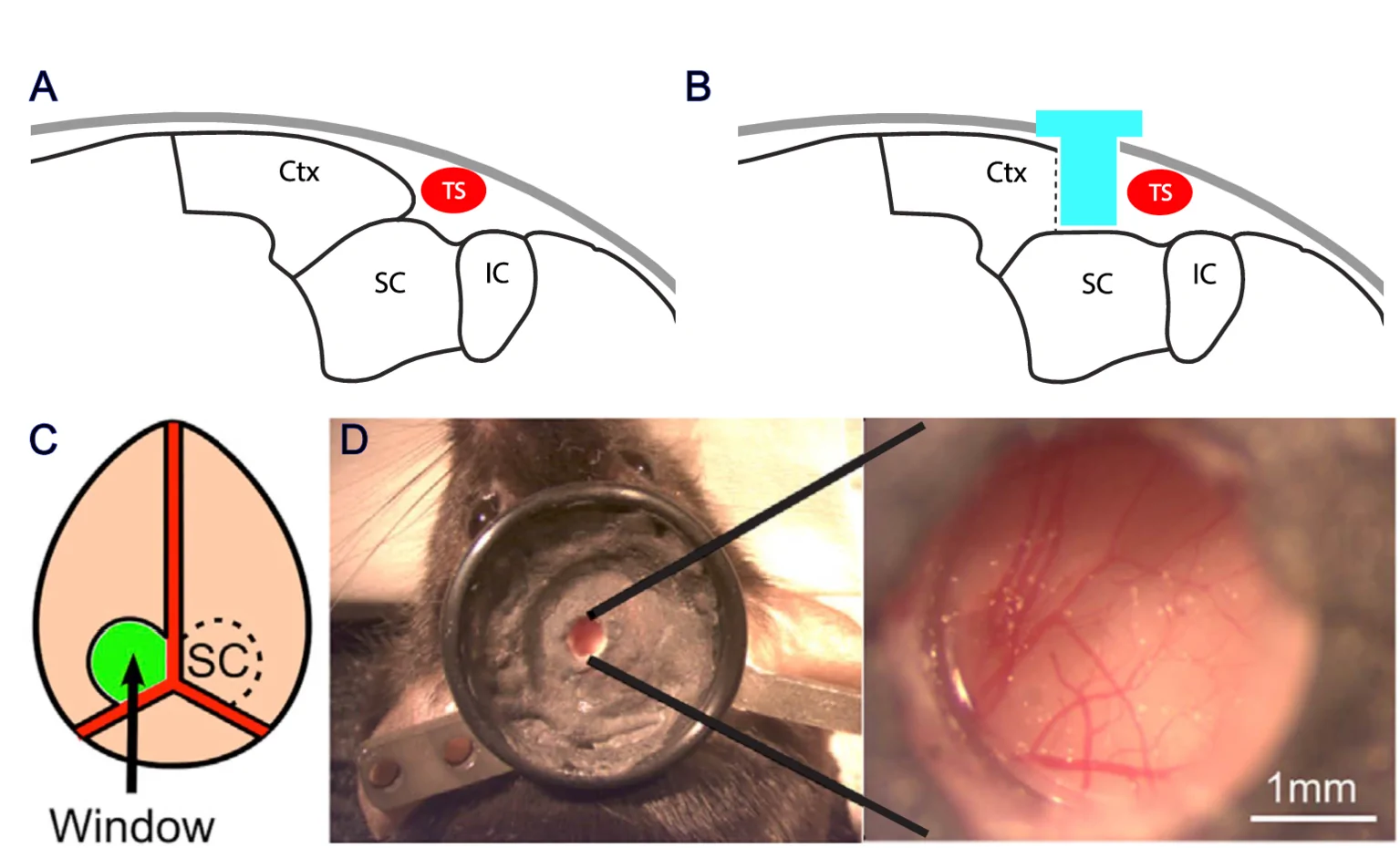
Surgical removal of the overlying visual cortex to access the SC. **(A)** Schematic sagittal view of the mouse superior colliculus (SC) and the surrounding brain regions (Ctx: cortex; IC: inferior colliculus). The skull is represented by a thick gray line, brain regions are delineated by black outlines, and the transverse sinus (TS) is shown in red. **(B)** The cortex overlying the SC is partially removed, and a glass assembly (cyan) is positioned above the exposed SC to provide optical access. Structures are not drawn to scale. Brain region outlines have been adapted from the Allen Reference Atlas – Mouse Brain, P56 sagittal atlas (Allen Institute for Brain Science, 2011). **(C)** Schematic view of the mouse brain from above showing the location of the imaging window used to provide optical access to the SC. Red lines indicate the sagittal sinus (vertical) and the transverse sinus (horizontal). **(D)** Photographs of the mouse head from above show the imaging window and craniotomy (left). A magnified view of the craniotomy and exposed SC after removal of the overlying visual cortex and implantation of the imaging window (right). Panels **(C,D)** were adapted from de Malmazet et al. (2018).

Removal of the overlying cortex exposes the anterior two-thirds of the SC, providing a relatively wide region for subsequent calcium imaging. This has been useful for performing population imaging and investigating the functional organization of the SC (de Malmazet et al., 2018; Inayat et al., 2015; Kasai and Isa, 2016, 2022). While the main advantage of this approach is that a large region of the SC is available for imaging, the major drawback is the need to remove the overlying visual cortex. This is an issue, as the mouse SC receives direct projections from the visual cortex, including from both the primary visual cortex (V1) and higher visual areas (Jiang et al., 2023; Wang and Burkhalter, 2013; Zhao et al., 2014). Furthermore, optogenetic silencing of the visual cortex has been shown to reduce stimulus-evoked responses in SC neurons by almost half while preserving their tuning properties (Zhao et al., 2014). The extent to which removal of this visual pathway impacts SC function, therefore, needs to be taken into consideration when using this approach. Recognizing this issue, some studies specifically indicate that efforts were made to minimize the volume of the cortex that was removed (Kasai and Isa, 2016, 2022).

## Method 2: Displacement of the transverse sinus

Blood vessels are typically avoided when performing *in vivo* two-photon calcium imaging, as blood absorbs and scatters both visible and infrared light, thereby acting as an optical barrier (Haiss et al., 2009). While the posterior (caudal) part of the SC does not have overlying cortex, it lies underneath a major horizontal blood vessel called the transverse sinus. An alternative method to gain optical access to the mouse SC involves displacing the transverse sinus and has been used in many recent imaging studies (Baruchin et al., 2023; Chen et al., 2021; Feinberg and Meister, 2015; Li and Meister, 2023; Li et al., 2020; Li Z. et al., 2023; Liang et al., 2023; Liu et al., 2023; Molotkov et al., 2023; Relota et al., 2025; Savier et al., 2019; Schroder et al., 2020). To the best of the authors’ knowledge, this method was first described by Feinberg and Meister (2015) while searching for a way to access the SC without removing the overlying cortex and thereby preserving the projection from the visual cortex to the SC. The transverse sinus lies in the dura and lacks a physical attachment to the brain surface, allowing it to be displaced without damaging the underlying SC. This is achieved by applying mechanical force parallel to the brain surface to displace the transverse sinus in the anterior direction, exposing the underlying SC for optical access (Feinberg and Meister, 2015).

With this method, an approximately 2.5–3-mm diameter craniotomy is first made over the lambda point. Notably, the bone is relatively porous in this area, so extra caution is required during the craniotomy procedure. Using a combination of a biopsy punch and a dental drill is useful during this step. Additionally, absorbable hemostatic gelatin sponges can be used to help control bleeding. Once the craniotomy is complete and the brain surface is exposed, a durotomy is performed posterior to the transverse sinus. A glass assembly is then placed on the dura-free part of the exposed SC. This glass assembly typically consists of triangular or rectangular glass pieces with a lateral size of approximately 1 mm^2^ and a thickness of 0.5 mm, glued onto the bottom of a round coverslip using ultraviolet (UV) curing glue (Chen et al., 2021; Liang et al., 2023; Liu et al., 2023; Relota et al., 2025; Savier et al., 2019). Instead of glass pieces, a metal tube (Baruchin et al., 2023; Schroder et al., 2020) or pre-cured transparent silicone (Feinberg and Meister, 2015; Li and Meister, 2023; Li et al., 2020) can also be used. Once the assembly is in position, it is pushed anteriorly to displace the transverse sinus approximately 0.5–1 mm, while pressure is applied downwards to ensure that the assembly remains in contact with the brain surface. Once in its final location, the assembly is secured with dental cement or cyanoacrylate glue (Figure 2).

**Figure 2:**
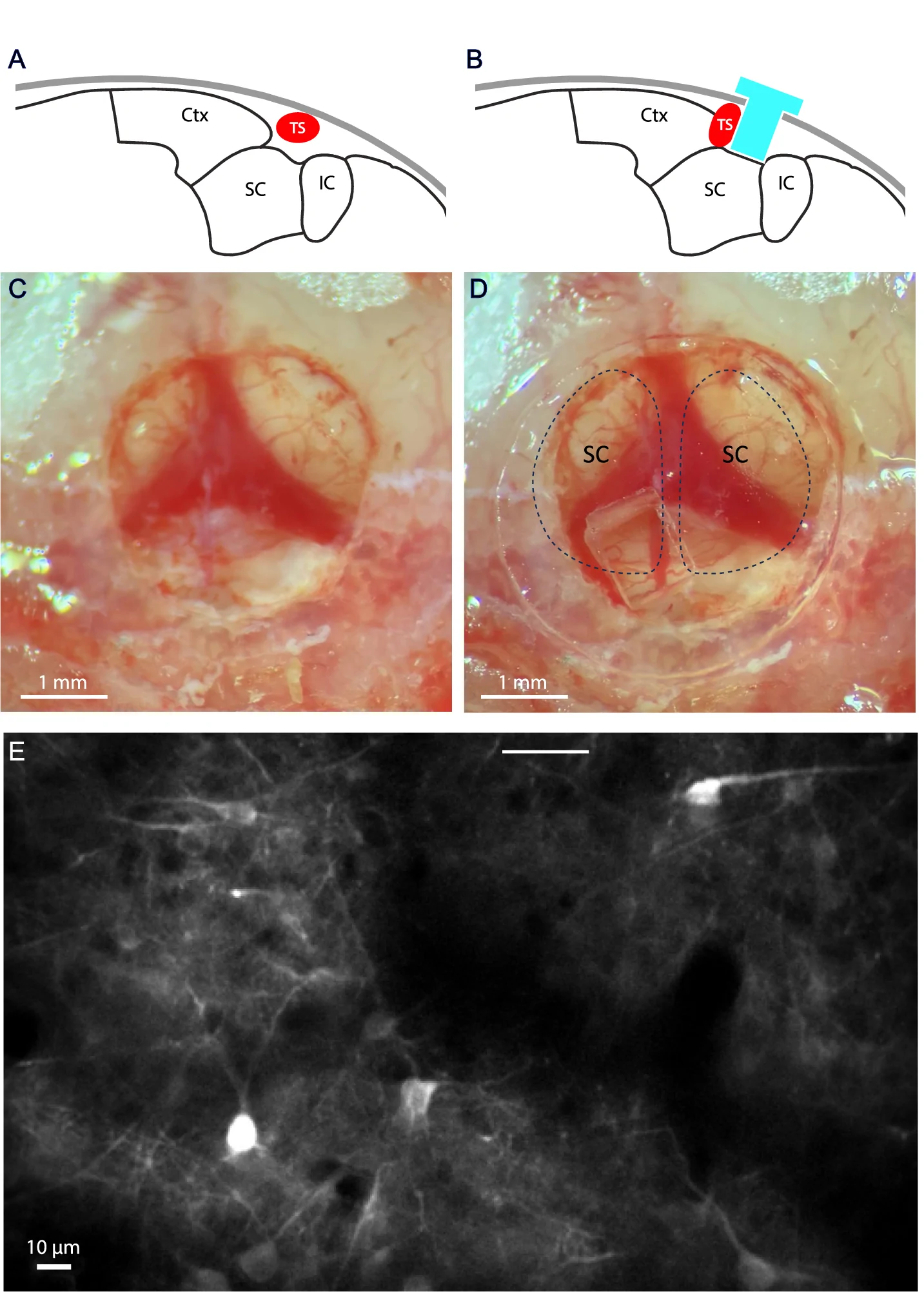
Surgical displacement of the transverse sinus using a glass assembly to enable two-photon imaging in the mouse SC. **(A)** Schematic sagittal view of a portion of the mouse brain and surrounding regions as described in Figure 1A. **(B)** The transverse sinus (TS) is displaced anteriorly by a glass assembly (cyan) positioned above the posterior SC, providing optical access while leaving the overlying cortex intact. Structures are not drawn to scale. Brain region outlines have been adapted from the Allen Reference Atlas – Mouse Brain, P56 sagittal atlas (Allen Institute for Brain Science, 2011). **(C)** Photograph of the craniotomy from above showing the sagittal sinus and the transverse sinus. Note that the region posterior to the transverse sinus is cloudy due to a thick layer of the dura. **(D)** Photograph showing the caudal end of the left SC after implantation of a multi-layered glass assembly, which was used to push the transverse sinus in the anterior direction. **(E)** Two-photon image of neurons expressing GCaMP7s in the mouse SC following surgical displacement of the transverse sinus. A Cre-dependent variant of GCaMP7s was virally injected into the SC of Ntsr1-cre mice for 4 weeks prior to imaging. The imaging depth was approximately 250 μm below the surface of the SC. Ctx, cortex; SC, superior colliculus; IC, inferior colliculus; TS, transverse sinus.

This approach has the major advantage of maintaining the overlying visual cortex and its projection to the SC. However, this comes at the cost of increased surgical complexity. The porous nature of the skull close to lambda makes performing a craniotomy in this region particularly difficult. In addition, displacement of the transverse sinus carries the risk of rupturing the sinus or its associated blood vessels, which may lead to uncontrolled bleeding. An additional disadvantage of this method is that only the posterior medial end of the SC can be imaged with this approach, which corresponds to approximately 15–25% of the surface area of the SC (Feinberg and Meister, 2015). When imaging the superficial layers of this region and performing visual experiments, it should be noted that this area corresponds to the upper lateral (temporal) visual field according to the retinotopic organization of the SC (Cang et al., 2018).

An alternative to displacing the transverse sinus in the anterior direction is to suture it and then remove the section of the transverse sinus overlying the SC. While more complicated, this method allows access to a larger region of the SC and can be used in combination with removal of the overlying cortex to allow optical access to the entire SC (Liang et al., 2023).

## Alternative approaches

In addition to surgical approaches for gaining optical access for two-photon calcium imaging, several alternative imaging strategies may prove useful for investigating SC function. One potential approach is to use three-photon imaging, which utilizes longer excitation wavelengths to achieve improved tissue penetration and reduced scattering, thereby enabling imaging of subcortical structures at depths of more than 1 mm below the brain surface (Fuhrmann et al., 2025; Horton et al., 2013; Ouzounov et al., 2017; Xu et al., 2024). In the case of the SC, this raises the possibility of performing imaging through the overlying visual cortex without the need to remove it. While three-photon imaging is technically demanding and typically has a slower imaging rate than two-photon imaging (Fuhrmann et al., 2025), recent advancements in spinning disk three-photon microscopy offer a promising approach for increasing imaging speed (Liao et al., 2024). Together with continued improvements in genetically encoded activity sensors, three-photon imaging may become a viable alternative for functional imaging of the entire SC without the need to remove the overlying cortex.

Another possible alternative is the use of gradient-index (GRIN) lenses, which act as an optical relay to provide optical access to deep brain regions with relatively limited disruption to overlying tissue. For the SC, this approach could enable calcium imaging via a GRIN lens implanted through the overlying visual cortex. While this method still requires an invasive surgical procedure and offers limited flexibility in imaging depth, as well as reduced spatial resolution compared to conventional two-photon microscopy, it provides an opportunity to investigate SC activity with minimal disruption to V1. In addition, it can also be combined with other imaging platforms such as miniaturized two-photon systems (Zong et al., 2022) or even three-photon imaging (Klioutchnikov et al., 2022).

Finally, tissue clearing represents another approach that could be explored in the future. This technique involves minimizing light scattering by introducing low-osmolarity, refractive index-matching polymers into the extracellular matrix and has recently been shown to increase tissue penetration when used in conjunction with *in vivo* two-photon imaging (Inagaki et al., 2026). In the context of SC imaging, optical clearing could be applied to the overlying visual cortex to improve optical access without the need for surgical intervention. Although achieving uniform clearing across the full cortical thickness above the SC is likely to be challenging, this approach has the potential to enable imaging of large areas of the SC using two-photon microscopy without disruption to V1.

## Discussion

By using the methods described above to gain optical access to the SC, together with genetically encoded calcium indicators or synthetic calcium sensors, *in vivo* two-photon calcium imaging studies have broadened our understanding of the functional properties and organization of SC neurons. These include orientation and direction selectivity (Chen et al., 2021; de Malmazet et al., 2018; de Malmazet et al., 2024; Feinberg and Meister, 2015; Li et al., 2020; Liang et al., 2023; Liu et al., 2023; Shi et al., 2017; Wang et al., 2010), pattern motion selectivity (DePiero et al., 2024), retinotopic organization (Inayat et al., 2015; Molotkov et al., 2023), and surround suppression (Kasai and Isa, 2016), as well as the effects of behavior, arousal, and reward on visual responses (Baruchin et al., 2023; Relota et al., 2025; Savier et al., 2019; Schroder et al., 2020). These studies have typically imaged SC neurons at depths of 150–400 μm below the SC surface, corresponding to the superficial layers of the SC, using laser powers ranging from 15 to 50 mW at the sample under the objective (Feinberg and Meister, 2015; Kuhn et al., 2025; Liang et al., 2023). While the majority of studies have focused on imaging the cell bodies of superficial SC neurons, some have also imaged subcellular structures such as the dendrites of SC neurons (Kuhn et al., 2025) or the axonal processes of retinal ganglion cells in the SC (de Malmazet et al., 2024; Molotkov et al., 2023; Schroder et al., 2020), which are more challenging to resolve due to their smaller diameter. Notably, the thickness of the superficial SC varies depending on its anterior-to-posterior or medial-to-lateral location, which may affect the ability of two-photon imaging to access different SC regions.

Despite these advancements in two-photon calcium imaging in the mouse SC, many questions remain to be addressed. For instance, the functional organization of the SC in terms of orientation and direction columns remains unresolved, with different groups reporting conflicting results (Chen et al., 2021; de Malmazet et al., 2018; de Malmazet et al., 2024; Feinberg and Meister, 2015; Inayat et al., 2015; Kasai and Isa, 2022; Li et al., 2020; Liang et al., 2023; Molotkov et al., 2023; Savier et al., 2019; Schroder et al., 2020). Furthermore, while there have been attempts to categorize functional cell types in the SC based on their visual responses using two-photon calcium imaging (Li and Meister, 2023), how different populations of SC neurons encode behaviorally relevant visual signals and drive appropriate behavioral responses remains poorly understood. Notably, even within genetically and morphologically defined cell classes in the SC, considerable diversity in response properties has been reported (Kuhn et al., 2025; Relota et al., 2025), suggesting the presence of functionally distinct subpopulations. Future two-photon imaging studies, in conjunction with advances in genetic tools, visual stimulation paradigms, and experimental approaches, will further broaden our understanding of signal processing in the mouse SC and the role of specific cell types in SC-mediated behaviors.
